# Tracking sex-dependent differences in a mouse model of CLN6-Batten disease

**DOI:** 10.1186/s13023-019-0994-8

**Published:** 2019-01-21

**Authors:** McKayla J. Poppens, Jacob T. Cain, Tyler B. Johnson, Katherine A. White, Samantha S. Davis, Rachel Laufmann, Alexander D. Kloth, Jill M. Weimer

**Affiliations:** 1 Pediatrics and Rare Diseases Group, Sanford Research, Sioux Falls, SD USA; 2 Department of Biology, Augustana University, Sioux Falls, SD USA; 3 Department of Pediatrics, Sanford School of Medicine, University of South Dakota, Sioux Falls, SD USA

**Keywords:** Neuronal ceroid lipofuscinoses, Rare disease, Lysosomal storage disorder, Neurodegenerative disease, Pediatric disease

## Abstract

**Background:**

CLN6-Batten disease is a rare neurodevelopmental disorder characterized pathologically by the accumulation of lysosomal storage material, glial activation and neurodegeneration, and phenotypically by loss of vision, motor coordination, and cognitive ability, with premature death occurring in the second decade of life. In this study, we investigate whether sex differences in a mouse model of CLN6-Batten disease impact disease onset and progression.

**Results:**

A number of noteworthy differences were observed including elevated accumulation of mitochondrial ATP synthase subunit C in the thalamus and cortex of female *Cln6* mutant mice at 2 months of age. Moreover, female mutant mice showed more severe behavioral deficits. Beginning at 9 months of age, female mice demonstrated learning and memory deficits and suffered a more severe decline in motor coordination. Further, compared to their male counterparts, female animals succumbed to the disease at a slightly younger age, indicating an accelerated disease progression. Conversely, males showed a marked increase in microglial activation at 6 months of age in the cortex relative to females.

**Conclusions:**

Thus, as female *Cln6* mutant mice exhibit cellular and behavioral deficits that precede similar pathologies in male mutant mice, our findings suggest the need for consideration of sex-based differences in CLN6 disease progression during development of preclinical and clinical studies.

**Electronic supplementary material:**

The online version of this article (10.1186/s13023-019-0994-8) contains supplementary material, which is available to authorized users.

## Background

Batten disease (neuronal ceroid lipofuscinoses) comprises a family of autosomal-recessive neurodegenerative diseases characterized by lysosomal accumulation of autofluorescent lipopigment [1–4]. Pathological hallmarks of Batten disease include neuronal death in cortical and thalamic regions of the brain and massive gliosis throughout the CNS, presenting functionally as degeneration of vision, psychomotor delay, and premature death [5–7]. CLN6-Batten disease, resulting from mutations in *CLN6*, constitutes two distinct diseases: a pediatric form, also referred to as variant late infantile neuronal ceroid lipofuscinoses, and a rare, less-severe adult-onset form referred to as Kufs type A disease [8, 9]. The pediatric variant of CLN6 disease begins between the ages of 18 months and 8 years, presenting with language impairment, motor deterioration and cognitive deficiencies, followed by vision loss, seizures, and ultimately premature death during the second decade of life [10]. There are a number of naturally occurring CLN6 animal models used in therapeutic development, including the *Cln6*^*nclf*^ mouse model that contains a similar point mutation as found in human patients and develops the classical pathophysiological hallmarks of Batten disease, such as intracellular inclusion, retinal degeneration, hind-limb paralysis, and premature death [11–13]. Many of the past Batten disease studies have excluded female mice from therapeutic studies to avoid confounding variables related to sex hormones and chromosomal differences [14, 15]. However, these biological disease modifiers can potentially limit the translatability of mouse findings to female patients, as sex-based differences that may affect disease susceptibility, disease severity, and therapeutic efficacy [14]. For example, sex specific symptomatic differences have been reported in other neurodegenerative diseases including Alzheimer’s disease, Parkinson’s disease, multiple sclerosis, and autism spectrum disorders [14, 16–19]. In addition, patient response to therapeutics has varied by sex, as estrogen, testosterone, and other sex-linked genes may affect drug effectiveness [20].

In the Batten disease field, studies on CLN3-Batten disease, a genetically distinct subtype of Batten disease, have shown differences in disease progression in patients depending on sex [8, 9, 21, 22]. On average, female CLN3-Batten disease patients present with symptoms 1 year later than their male counterparts, have accelerated disease progression following symptom onset, and die 1 year earlier than males [21]. Initial characterization of the *Cln3*^*Δ7/8*^ murine model did not consider sex-based differences, however, recent work with this model has demonstrated that females exhibit poorer performance in behavioral tests [23, 24]. Additionally, the naturally occurring mouse model of CLN8-Batten disease has shown sex differences in female *Cln8*^*mnd*^ mice, where female retinas exhibited higher levels of retinal oxidative stress and caspase-3 activity compared to males [25]. These findings prompted us to investigate sex discrepancies in outcomes associated with CLN6 disease, exploring differences in disease onset and progression between male and female *Cln6*^*nclf*^ mice. We describe subtle histopathological differences between the sexes and a more rapid disease progression in female *Cln6*^*nclf*^ mice. Consequently, including sex as a factor during studies and subsequent analyses can ensure proper development of therapeutic treatments for patients with CLN6 disease.

## Results

### *Cln6*^*nclf*^ mice have sex and age dependent pathological differences in the brain

**Fig. 1:**
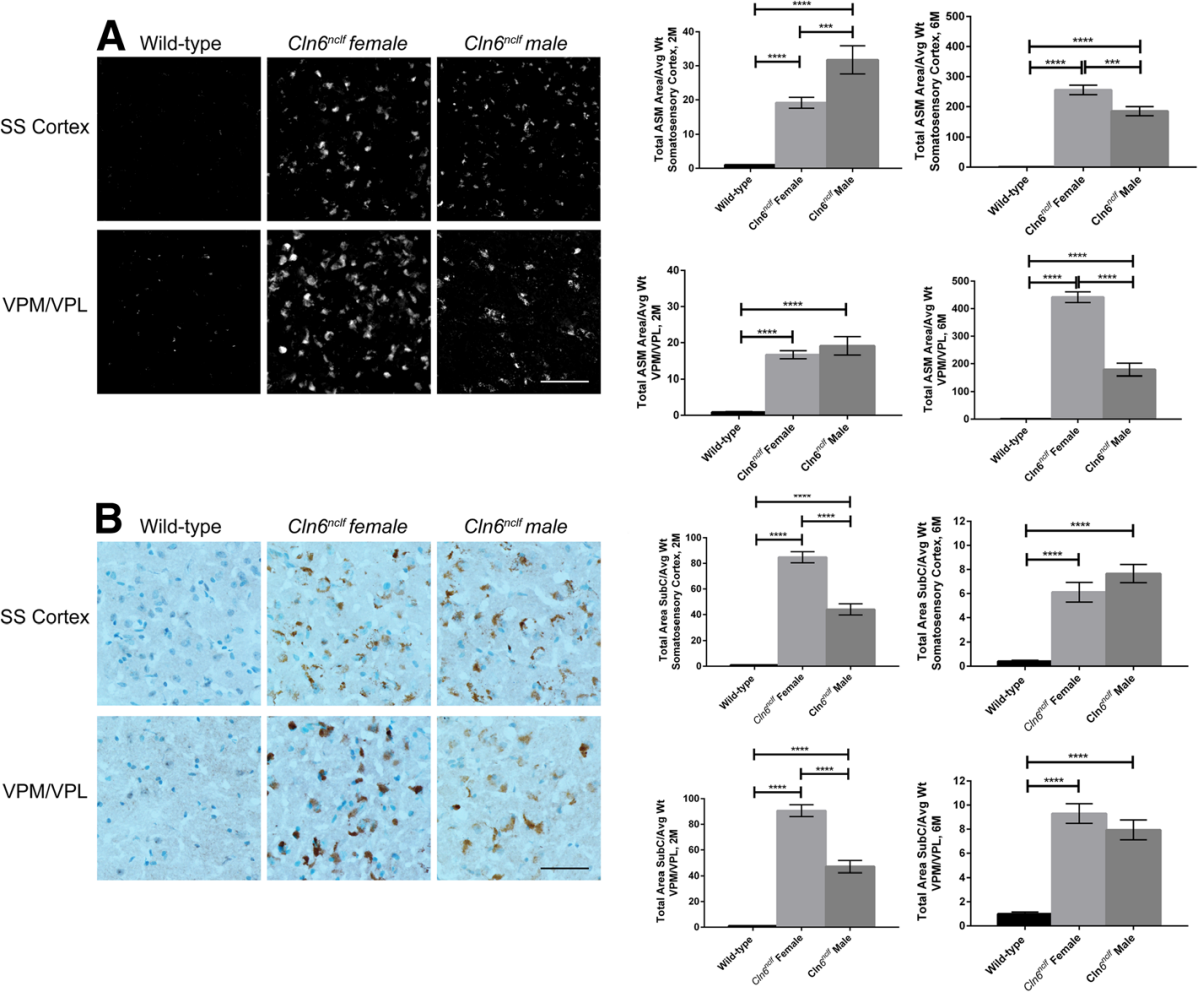
Sex differences evident in *Cln6*^*nclf*^ accumulation of autofluorescent storage material (ASM) and mitochondrial ATP synthase subunit C in brain. **a** Male *Cln6*^*nclf*^ mice show enhanced ASM in the somatosensory cortex at two months of age, while female *Cln6*^*nclf*^ mice overtake their male counterparts at six months of age in the VPM/VPL and somatosensory cortex. **b** Female mice show enhanced subunit C expression at two months of age, that corrects as the animals age to six month. Images represent the six month time point only. *N* = 3–5, ***p < 0.001, ****p < 0.0001

**Fig. 2:**
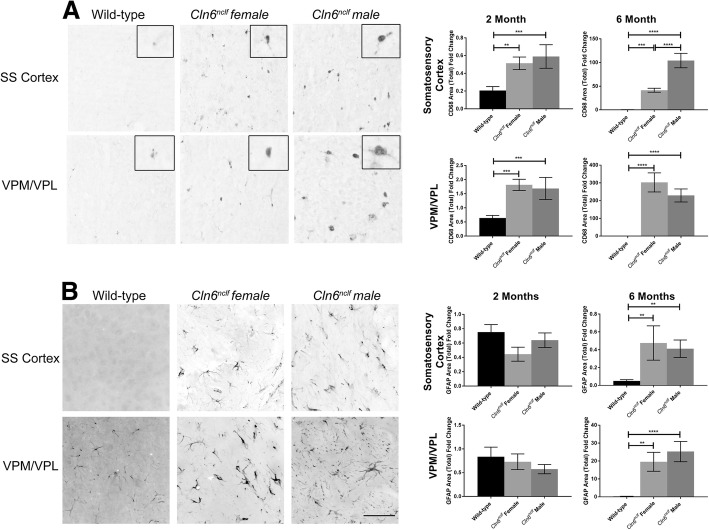
Sex differences evident in *Cln6*^nclf^ glial activation in brain. **a** Male *Cln6*^*nclf*^ mice show enhanced microglial expression (CD68) in the somatosensory cortex at six months of age. **b** Genotypic differences in astrocyte activation are not present until six months of age, and are similar between the sexes. Images represent the six month time point only. *N* = 4–6, *p < 0.05, ***p < 0.001, ****p < 0.0001

Sex dependent differences in the classic pathological hallmarks of Batten disease were examined in the thalamus and somatosensory cortex of wild-type and *Cln6*^*nclf*^ mice, two areas of the brain that are affected early in Batten disease. Accumulation of autofluorescent storage material (ASM) in the brain is a manifestation common to all variants of Batten disease. At two and 6 months of age, *Cln6*^*nclf*^ mice of both sexes had accumulation of ASM within the ventral posteromedial and ventral posterolateral (VPM/VPL) nuclei of the thalamus and somatosensory cortex relative to wild-type mice (Fig. 1a, b). At all-time points examined and within each brain region examined, wild-type males versus females showed no difference from one another (data not shown) and, therefore, are represented as a single combined sample. Male *Cln6*^*nclf*^ mice had significantly more ASM in the somatosensory cortex at 2 months than their female counterparts (30 fold increase), however at 6 months the females had increased levels ASM in both regions (300 to 450 fold increase). This possibly reflects the observation reported in CLN3-Batten disease patients females present with a faster disease progression [21]. As an additional measure of cellular accumulation, mitochondrial ATP synthase subunit C, a constituent of ASM, was examined as well. While *Cln6*^*nclf*^ mice exhibited greater accumulation of subunit C in the VPM/VPL and somatosensory cortex at both time points, female *Cln6*^*nclf*^ mice showed greater subunit C burden than male *Cln6*^*nclf*^ mice at 2 months of age (80 fold increase) (Fig. 2c, d). By 6 months of age, this difference had leveled off between the sexes (10 fold increase). Considering 6 month female *Cln6*^*nclf*^ mice accumulate greater amount of total ASM, it’s possible that this accumulation is made up of constituents other than subunit C.

Reactive gliosis is another marker of Batten disease that can be used to measure disease severity as the mice age. At 6 months of age, while *Cln6*^*nclf*^ mice collectively had elevated astrocyte activation (GFAP^+^) and microgliosis (CD68^+^) in the VPM/VPL and somatosensory cortex, there were no differences between the sexes (0.5 to 20 fold increase) (Fig. 2a-d). Interestingly, male *Cln6*^*nclf*^ mice had heightened microgliosis in the somatosensory cortex compared to their female *Cln6*^*nclf*^ counterparts at 6 months of age (100 fold increase) (Fig. 2b). As glial activation can contribute to or ward off neuron loss, we also assessed whether there was any gross neuronal loss between the sexes at these time points. When measuring the thickness of the cortical plate in several regions, there were no differences between wild-type and *Cln6*^*nclf*^ mice at any time point in any region (Additional file 1: Figure S1). Thus, any changes in classic Batten disease pathology did not provoke a gross loss or stabilization of neurons in either sex up to 6 months of age.

### *Cln6*^*nclf*^ mice have genetic, sex, and age dependent differences in behavioral tests

**Fig. 3:**
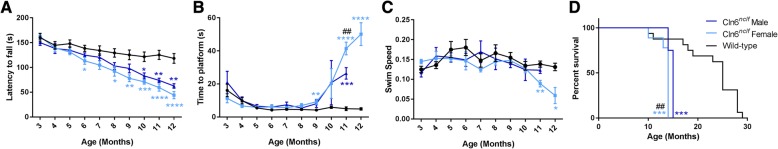
Sex differences evident in *Cln6*^*nclf*^ behavior and survival outcomes. **a** Female *Cln6*^*nclf*^ mice perform more poorly at the rotarod motor task beginning at six months of age. Male *Cln6*^*nclf*^ mice do not begin to perform poorly until 10 months of age. **b** Female *Cln6*^*nclf*^ mice perform more poorly at the Morris water maze task beginning at nine months of age. Male *Cln6*^*nclf*^ mice do not begin to perform poorly until 11 months of age. **c** Swim speed shown as a control for the Morris water maze task. **d** Female *Cln6*^*nclf*^ mice perish one month earlier than male *Cln6*^*nclf*^ mice. Asterisks (*) show comparisons between wild-type and *Cln6*^*nclf*^ animals, with light blue for female comparisons and dark blue for male comparisons. Hash signs (#) show comparisons between male and female *Cln6*^*nclf*^ animals. *N* = 3–10, *p < 0.05, **p < 0.01, ***p < 0.001, ****p < 0.0001

Next, we examined whether there were sex differences in *Cln6* mutant mice in neurobehavior performance and long term survival. As a measure of motor coordination and balance, along with motor learning aptitudes and endurance, we tested mice on the rotarod test beginning at 3 months of age. Again, no differences were detected between sexes in wild-type mice on any of the assay performed, thus are represented as one combined sample. Prior to 6 months of age, wild-type and *Cln6*^*nclf*^ animals maintain relatively similar latency times. At 6 months, the *Cln6*^*nclf*^ female mice began to show deficits in motor performance, while diminished ability did not become apparent in male mice until after 10 months of age (Fig. 3a). Deficits became more prominent in both sexes over time.

In the Morris water maze, a navigation test of spatial learning and memory, *Cln6*^*nclf*^ mice required more time than wild-type mice to navigate to the platform beginning at 11 months of age, while female *Cln6*^*nclf*^ mice specifically showed performance regression as early as 9 months of age (Fig. 3b). Importantly, at 11 months, female *Cln6*^*nclf*^ mice reached the platform later than male *Cln6*^*nclf*^ mice, indicating that female *Cln6*^*nclf*^ mice have more prominent learning and memory deficits than male mice of the same age. Female *Cln6*^*nclf*^ mice continued to perform poorly at 12 months of age, but could not be compared to *Cln6*^*nclf*^ male mice at this age as the male mice were unable swim, due to physical conditions. Swim speed is shown as a control, and it should be noted that 11- and 12-month-old female *Cln6*^*nclf*^ mice were slower than their wild-type counterparts in reaching the platform. A slower swim speed may have been a confounding variable in the analysis of navigation times of the animals at 11 and 12 months.

Lastly, we plotted a Kaplan-Meier survival curve to estimate the fraction of living *Cln6*^*nclf*^ animals over time. Diseased animals’ premature death occurred 1 month earlier, on average, for female *Cln6*^*nclf*^ mice compared to their male counterparts (14 and 15 months, respectively), while wild-type mice lived to ~ 28 months (Fig. 3c).

## Discussion

In this study, we show differences in the progression CLN6 disease between sexes in a *Cln6*^*nclf*^ mouse model. Female *Cln6*^*nclf*^ mice presented with an earlier increase subunit C accumulation, and subsequently performed more poorly on behavioral tests and perished earlier than their male counterparts. Male *Cln6*^*nclf*^ mice, on the other hand, showed an increase in ASM at an earlier time point, as well as an increase in microglial activity at 6 months of age. While there have been previous comprehensive natural history studies of CLN6 disease patients, interpretation of sex-based outcomes is limited due to varying *CLN6* mutations [9, 26, 27]. In the CLN3 variant of Batten disease, where the delta 7/8 mutation is common and affects ~ 75% of patients, male patients display symptoms before female patients, though females ultimately progress more quickly and die prior to males [21, 28]. It’s possible that the momentary increase in ASM seen in male mice reflects an early disease presentation, though this doesn’t translate into earlier functional difficulties. As the molecular underpinnings of CLN6 disease are not well understood, the extent to which these pathological changes translate to behavior changes in *Cln6* mice will need to be the subject of future study.

The observed sex driven differences in disease progression are not unique to the *Cln6*^*nclf*^ mice or Batten disease. While the biological basis for variance in disease progression between sexes is unknown, hormonal factors may contribute to the observed differences. Among adults with neurodegenerative diseases, estradiol appears to play a protective role in females [29, 30]. However, in adolescent females with juvenile Batten disease, estrogen may be doing the exact opposite: CLN3-Batten disease females of post-pubertal age, when estrogen levels are elevated, demonstrated earlier loss of independence, and thus, estrogen may be contributing to the rapid disease progression [21].

Batten disease is an immune-mediated disease characterized by chronic neuroinflammation that is sustained by persistent glial activation in the brain, leading to damage and death of neighboring neurons and glial cells. Gonadal hormones support coordination of neuron-glia interactions and regulate reactive gliosis and neuroinflammation [31–33]. Control of reactive gliosis by progesterone and estradiol is well documented, yet gliosis regulation by androgens has not been extensively explored. In general, estradiol appears to reduce astrocyte activity in the cerebral cortex, though, this contradicts the female *Cln6* mouse presentation of heightened astrocytosis. However, evidence suggests that testosterone decreases reactive astroglia and microglia after neuronal damage [31, 32, 34]. Further, female microglia have been shown to exhibit higher phagocytic capacity than males and proinflammatory conditions, while male microglia have more efficient migratory response [35, 36]. Because glial cells become cytotoxic when chronically activated, female reduction in microglial activity may be a sign of advanced disease progression as these glial cells may have become inactive [37]. Furthermore, sex differences in microglial number may result from differences in chemotactic signaling and consequent microglial recruitment in males and females. Indeed, males exhibit higher levels of chemokines CCL20 and CCL4 in the hippocampus and cortex during critical periods of development, while females have elevated levels of proinflammatory cytokine interleukin (IL)-1β [38]. However, chemokines have not been studied in great detail in a healthy brain or in Batten disease, and the extent to which they may play a part in neurodegeneration remains unclear. Additionally, females are more vulnerable than males to develop Alzheimer’s disease, and although androgens have been studied less extensively than estrogens, androgens exert anti-inflammatory effects on microglia in AD models [39, 40].

Sex-based differences in Batten disease may also be related to the rise of autoantibodies in females. Estrogen has been shown to increase autoantibodies in systemic lupus erythematosus, accelerating disease progression [41]. In Batten disease, a similar autoimmune response in the CNS of both animal and patient populations contributes to disease progression [21]. Overall, hormonal differences in males and females likely explain some sex-specific immune responses, modifying disease course. As suppression of the immune system has been used in preclinical and clinical Batten disease studies, the extent to which these therapies are beneficial in both sexes should be a point of focus in the future [42, 43].

## Conclusions

Here, we provide the first sex comparison of pathological and behavioral differences in *Cln6*^*nclf*^ mice, finding notable differences between the sexes. Moreover, our findings are identical to observed by another laboratory working with the same *CLN6* mutant strain (personal communication, Drs. Stephanie Hughes and Hannah Best). The *Cln6*^*nclf*^ mouse model echoes the accelerated disease progression reported of females with Batten disease, and this information will be instrumental in providing appropriate treatments to female Batten disease patients in the future [21]. Currently, no definitive treatments or cures exist for CLN6 disease, a rapidly-progressing neurodevelopmental disease. However, as potential therapeutics are being investigated, sex-related differences must be taken in to account to target translatability to women.

## Methods

### Ethics statement/animals

All animal studies were performed in an AAALAC accredited facility in strict accordance with National Institutes of Health guidelines and were approved by the Sanford Institutional Animal Care and Use Committee (USDA License 46-R-0009). Wild-type and homozygous *Cln6*^*nclf*^ mutant mice (Jackson Laboratory, Bar Harbor, ME) on C57BL/6 J backgrounds were used for all studies and were housed under identical conditions. For the immunohistochemistry experiments, 3–6 mice were used per group. For the behavior studies, 10 mice were used per group. As the mice aged and perished, this reduced the N in some groups to 3 at the last few time points.

### Neurobehavior testing

#### Rotarod

Beginning at 3 months of age, mice were tested monthly (up to 12 months of age) on a Rotamex-5 Rotarod (Columbus Instruments, Columbus, OH, USA) to assess motor abilities. The machine’s initial speed was set to 0.3 rpm (rpm) and accelerated at 0.3 rpm every two seconds until maximum speed (36 rpm) was reached. Mice were trained over 9 trials: 3 consecutive trials, followed by a 30-mintue resting period, 3 more consecutive trials, followed by another 30-min resting period, and a final 3 consecutive trials. Subsequent testing after a four-hour resting period modeled the training session. Latency time to fall from the rod was recorded and averaged for each of a mouse’s nine testing trials to give one value per mouse.

#### Morris water maze

Mice were tested monthly (from months 3–12) using a standard Morris water maze protocol to assess memory and learning deficiencies. A 4-ft diameter tub was filled with water (about 26 in. in depth) and a goal platform was placed 0.5 cm below the water’s surface. Four visual cues surrounded the tub at 0 (N), 90 (E), 180 (S), and 270 (W) degrees; the platform was set at 315 (NW) degrees in the maze. Mice were initially trained in the tub with clear water and a flagged platform. Mice were given 60 s per trial for eight trials to locate the platform; four trials in the morning, a three-hour resting period, and four additional trials in the afternoon. Mice unable to locate the platform with 50% accuracy in the allotted time were eliminated from testing. The remaining mice were then tested in opaque water colored with white non-toxic tempura paint and an unflagged platform. On each test day, the mice were given 60 s per trial for eight trials to locate the platform; a training session consisting of four trials was implemented in the morning followed by a three-hour resting period, followed by a testing session of four trials in the afternoon. Mice were tested on four consecutive days, starting at a different visual cue each day. Any-maze software (Stoelting Co., Wood Dale, IL, USA) tracked test duration and swim speed for each mouse. Quantifications of each recording were averaged from the sixteen afternoon trials per mouse.

### Immunohistochemistry

Wild-type and *Cln6*^*nclf*^ mice were CO_2_ euthanized, perfused with PBS, and tissue fixed with 4% PFA. Fixed brains were sectioned on a vibratome at 50 μm (Leica VT10008) and processed with standard immunofluorescence and DAB staining protocols as previously described [44]. Primary antibodies included anti-CD68 (AbD Serotec, MCA1957; 1:250), anti-GFAP (Dako, Z0334; 1:250), and anti-ATP synthase subunit C (Abcam, ab181243, 1:500). The subunit C experiments were also counterstained with methyl green. Secondary antibodies included anti-rat and anti-rabbit biotinylated (Vector Labs, BA-9400; 1:2000) and Alexa-Fluor fluorescent secondaries (1:1500). Sections were imaged in the VPM/VPL of the thalamus and layers 2/3 of the somatosensory cortex and analyzed using a Nikon 90i microscope with NIS-Elements Advanced Research software (v4.20). For autofluorescent storage material, cells were scored positive for accumulation of storage material when more than three autofluorescent puncta were aggregated around the nucleus. Mitochondrial ATP synthase subunit C, GFAP, and CD68 immunoreactivity was quantified using a threshold analysis in NIS-Elements Advanced Research software, with the subunit C analyzed with the methyl green counterstain excluded from analysis (v4.20) as previously described [44].

#### Cortical plate thickness

Cortical plate thickness was measured in the visual, motor, and somatosensory cortex of sagittal tissue sections. Measurements were taken in triplicates in the cortical plate, encompassing layers 1–6 of the cerebral cortex. Triplicates were averaged, and statistical tests performed as described.

### Statistical analyses

Statistical analyses were performed using GraphPad Prism (v6.04). Equal numbers of male and female wild-type animals were combined into one group, as there were no differences between male and female wild-type values for any given assay. For immunohistochemical analyses, one-way ANOVA’s were utilized with Tukey correction and outlier removal using the ROUT method, Q = 1. One-way ANOVA’s with Tukey correction and outlier removal using the ROUT method, Q = 1, were also used for behavior experimentation analyses. For the Morris water maze 12-month timepoint, an unpaired t-test was used. To develop a survival curve, the log-rank (Mantel-Cox) test was used.

## Additional file

Additional file 1: **Figure S1.** No gross cortical neuron loss detected at 2 or 6 months of age in *Cln6*^*nclf*^ mice. Mean +/− SEM. N = 3–6. (TIF 9028 kb)
