# Supplementary figures and images for: Tracking sex-dependent differences in a mouse model of CLN6-Batten disease
Source: Orphanet J Rare Dis. 2019 Jan 21;14:19. doi: 10.1186/s13023-019-0994-8 (PMC6341540; doi:10.1186/s13023-019-0994-8)

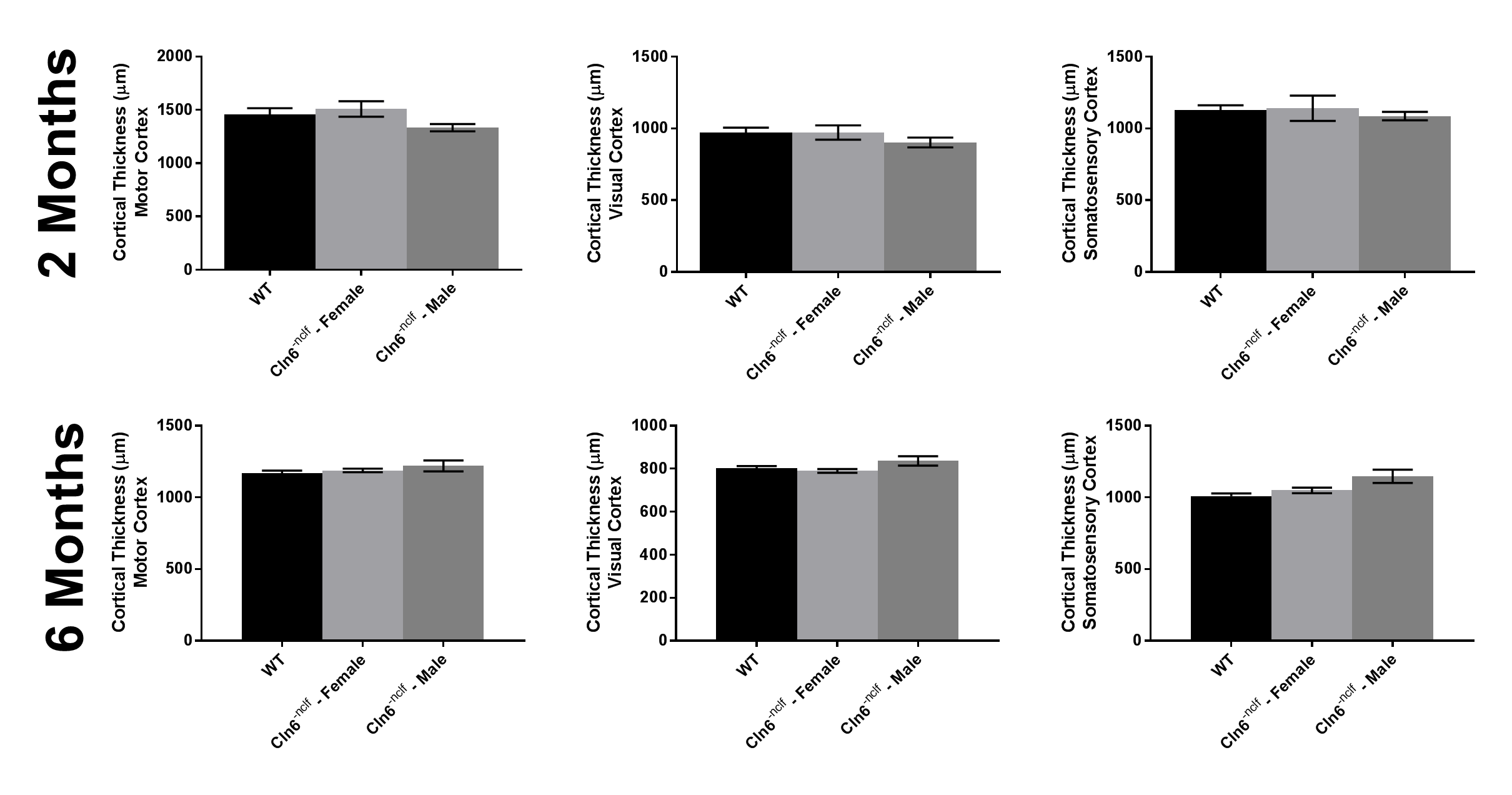

Supplement: Supplementary file 1 — Figure S1. No gross cortical neuron loss detected at 2 or 6 months of age in Cln6nclf mice. Mean +/− SEM. N = 3–6. (TIF 9028 kb) [file 13023_2019_994_MOESM1_ESM.tif]
